# Dose-response curves for the effects of eucalyptus essential oil nanoemulsion on growth performance and health status of heat-stressed growing rabbits

**DOI:** 10.5713/ab.25.0073

**Published:** 2025-07-11

**Authors:** Fatma Mohsen Shalaby, Soha A. Hassan, Salma K. Attia, Amany Omar Elrefaie, Ali Ali EL-Raghi, Kandil Abd El Hai Attia

**Affiliations:** 1 Department of Biology, Faculty of Sciences, King Khalid University, Abha, Kingdom of Saudi Arabia; 2 Department of Biotechnology, Faculty of Applied Health Sciences Technology, October 6 University, October 6 City, Egypt; 3 Faculty of Medicine, Mansoura University, Mansoura, Egypt; 4 Department of Pathology, Faculty of Medicine, King Khalid University, Abha, Kingdom of Saudi Arabia; 5 Department of Animal, Poultry and Fish Production, Faculty of Agriculture, Damietta University, Damietta, Egypt; 6 Evaluation of Natural Resources Department, Environmental Studies and Research Institute, University of Sadat City, Minufiya, Egypt

**Keywords:** Essential Oil, Health Status, Heat Stress, Nanoemulsion, Rabbit

## Abstract

**Objective:**

Dose-response curves were performed to explore the effect of eucalyptus essential oil nanoemulsion (EEONE) on the growth rate, feed efficiency, and health status of growing rabbits facing heat stress.

**Methods:**

Sixty growing rabbits (6 weeks old), were randomly divided into four homogeneous groups, receiving diets supplements with 0, 100, 200, and 400 mg EEONE/kg diet (EEONE0, EEONE100, EEONE200, and EEONE400, respectively).

**Results:**

Significant improvements were observed in growth performance, feed utilization, and physiological responses in the EEONE-treated groups compared to the control (p<0.05). Regression analysis revealed quadratic increases in red blood corpuscles and quadratic decreases in white blood cell counts in response to EEONE treatment, with optimal concentrations showed at 325 and 300 mg EEONE, respectively. Hemoglobin concentration, platelet count, blood protein and glucose levels linearly increased, while liver enzymes decreased significantly due to the EEONE treatment. The aberrant changes observed in liver tissues due to heat stress were effectively reversed, leading to a restoration of hepatic morphology closely resembling normal conditions, by co-administration of EEONE. Serum urea and creatinine concentrations decreased quadratically in the EEONE-treated groups, minimizing at 300 mg EEONE. Quadratic regression analysis indicated that the optimal doses were 300 mg EEONE for glutathione peroxidase and protein carbonyl, and 350 mg EEONE for total antioxidant capacity and malondialdehyde. Cellular and humoral immunity as well as pro inflammatory cytokinase significantly improved by the dietary treatment.

**Conclusion:**

Supplementing the diet with EEONE at levels of 300–400 mg can effectively improve growth metrics and health status of fattened rabbits.

## INTRODUCTION

Rabbits are primarily raised for meat, fur, and hair. Their meat is nutritious, being low in cholesterol, fat, and sodium, and high in healthy fats, protein, minerals, and vitamins [1]. Global warming and climate change have intensified heat stress (HS), posing a major challenge for the rabbit industry, especially in hot regions. Rising temperatures make HS the most severe stressor in rabbit and poultry farming [2]. Rabbits are more vulnerable to HS compared to other livestock sectors due to their dense fur coat and limited sweat glands, complicating heat dissipation mechanisms [3]. Moreover, genetically enhanced rabbits with higher metabolic activity and accelerated growth rates are more prone to HS, resulting in substantial economic losses in rabbit production. HS disrupts normal physiological functions, raises body temperature, and impairs growth performance, meat quality, reproductive performance, redox balance, and immune response in rabbits [4]. There is a rising interest in natural alternatives to antibiotics within rabbit production, aiming for antibiotic-free rabbit products. Phytogenics or phytobiotics are gaining popularity in rabbit nutrition for their roles as physiological stimulants, antioxidants, colorants, flavor enhancers, digestive aids, and in treating various health issues [5,6]. Notably, former studies highlight the untapped potential of eucalyptus globulus leaves, rich in polyphenolic compounds with significant antibacterial, antioxidant, and anti-inflammatory features. The essential oil extracted from eucalyptus presents promising health benefits, offering a valuable avenue for combatting diverse ailments [7].

The eucalyptus essential oil (EEO) contains several bioactive compounds like 1,8-cineole, p-cymene, spathulenol, α-phellandrene, cryptone aldehydes, β-phellandrene, phellandral, and cuminal, providing anti-inflammatory, anti-oxidative, and antimicrobial benefits [8]. Moataz et al [5] demonstrated that EEO can improve the growth performance and health status of growing rabbits exposed to HS. However, the use of essential oils as dietary feed additives is limited due to challenges such as low permeability, bioavailability, solubility, and storage stability. Several studies have demonstrated that formulating essential oils into nanoemulsions enhances their biological efficacy, including antioxidant, anti-inflammatory, and antimicrobial properaties by improving the bioavailability and cellular uptake of bioactive constituents. Importantly, some of these effects have been confirmed in rabbit models or through studies involving nano-formulated essential oils [3], supporting their potential use in rabbit production systems. Based on these insights, this study aimed to assess the dose-dependent effects of dietary eucalyptus essential oil nanoemulsion (EEONE) supplementation on growth performance, physiological responses, and overall health in growing rabbits subjected to HS.

## MATERIALS AND METHODS

The present study was carried out at a privately owned rabbit farm located in Mit Ali, a suburban area of El-Mansoura City, Dakahlia Governorate, Egypt, located at approximately 31.032°N latitude and 31.378°E longitude.

### Preparation of eucalyptus essential oil nanoemulsion

EEO was sourced from Pure Life Company, Giza, Egypt. To prepare a monolayer nanoemulsion, 20% EEO was gradually emulsified with approximately 68 mL of water containing 10% surfactant (Tween 80) and 2% co-surfactant (ethanol), under gentle stirring at 25°C. The water was added at a controlled rate of 1.0 mL/min. The resulting emulsion underwent ultrasonic treatment in a water bath for 30 minutes, followed by further homogenization using an ultrasonic probe (Model VC 505; Sonics Vibra-Cell) set at 60% amplitude with a 1-second ON/1-second OFF pulsing cycle for 5 minutes. The final nanoemulsion was produced with an oil-to-surfactant ratio of 2:1.

### Physicochemical properties of eucalyptus essential oil nanoemulsion

The internal morphology of the freshly prepared EEONE was visualized using a transmission electron microscope (TEM) operating at 200 kV (TEM, JEM-2100; JEOL). The analysis of captured images was performed using Digital Micrograph and Soft Imaging Viewer software (ver. 2.11.1404.0; Gatan Microscopy Suite Software).

### Experimental design

Sixty weaned V-line male rabbits, aged 6 weeks with an initial body weight of 694.85±5.65 g, were evenly divided into four groups. The control group received a diet without EEONE, while the other three groups were provided the same diet supplemented with 100, 200, and 400 mg EEONE/kg diet (termed EEONE100, EEONE200, and EEONE400, respectively). This dietary regimen spanned 8 consecutive weeks during summer season.

### Experimental animals, management, housing and diets

The rabbits were housed in galvanized wire battery cages (measuring 50×50×40 cm) under a 12-hour dark - light cycle. They were fed a nutritionally balanced diet according to the guidelines by [9] as outlined in Table 1 with access to fresh water and pelleted rations *ad libitum* via automatic waterers and feeders. The facility was naturally ventilated and maintained high hygiene and management standards. To ensure even distribution, EEONE were mixed with 1 kg of diet before being uniformly blended with the rest. Throughout the study period, live body weights were recorded at the initial and end, and average body weight gain calculated. Feed consuming was meticulously monitored and quantified in grams/rabbit/week. Daily feed residuals were weighed, and factored into intake calculations. The feed conversion ratio (FCR) was determined as feed intake (in grams) per gram of live body weight gained. The performance index (PI) was calculated as:


(1) PI (%)=(Final live body weight [Kg]×100)/FCR.

### Microclimatic data

Within the Rabbitry, indoor climatic conditions were monitored daily at 8:00 am and 2:00 pm for ambient temperature (AT, °C) and relative humidity (RH, %) using an electronic digital thermo-hygrometer. The correlation between RH and AT was quantified as the temperature-humidity index (THI), calculated according to the equation established by [10].


(2) $$\text{THI}=\text{t}-\left[\left(0.31-0.31\left(\frac{\text{RH}}{100}\right)\right)\times (\text{t}-14.4)\right]$$

Here, ‘t’ is the dry bulb temperature in degrees Celsius, and ‘RH’ represents the relative humidity. THI values below 27.8 indicated the absence of HS, while values in the range of 27.8 to 28.9 denoted moderate HS. THI values between 29.0 and 30.0 were indicative of HS, while values exceeding 30.0 suggested very severe HS.

### Physiological responses

During the experiment period, the rectal temperature and respiratory rate were assessed biweekly. The respiratory rate was determined with a stopwatch by counting the number of flank motions for each rabbit per minute while the animals were at rest. Rectal temperatures were measured using a digital thermometer inserted 4 cm into each rabbit’s rectum for precise readings in Celsius (°C).

### Slaughtering and carcass characteristics

Five male rabbits were randomly selected from each group at the end of study period. These rabbits underwent a 12-hour fasting period, were individually weighed, and then humanely slaughtered in accordance with Islamic guidelines, ensuring the use of a sharp knife wielded by a qualified person, with specific prayers spoken during the process. After exsanguination, the pelt, viscera, and tail were carefully removed, and the carcass along with its various parts were weighed. To ascertain the dressing percentage, the weight of the hot-dressed carcass was divided by the pre-slaughter weight.

### Determination of biochemical parameters

As part of the slaughtering process, 5 mL of blood was extracted from each rabbit and subsequently classified as two subsamples for distinct analyses. The initial subsample was treated with an anticoagulant to assess hematological characteristics using an automated hematology analyzer (Hospitex Hema Screen 18). The second subsample was allowed to coagulate, and then centrifuged at 3,500 rpm for 15 minutes to separate serum samples. These serum samples were then preserved at −20°C until further examination. Various biochemical blood parameters, such as plasma total protein, albumin, triglycerides, total cholesterol, alanine aminotransferase, aspartate aminotransferase, and glucose, were analyzed using colorimetric assays with commercial kits procured from Bio-diagnostic, Cairo, Egypt, according to manufacturers’ guidelines. To assess antioxidant parameters, including total antioxidant capacity (TAC), superoxide dismutase (SOD), glutathione, and glutathione peroxidase (GSH-Px), quantitative sandwich ELISA kits were utilized following the recommended protocols. Furthermore, levels of protein carbonyl (PC) and malondialdehyde (MDA), indicative of protein and lipid oxidation, were measured using specialized ELISA kits (MDA: MBS8806802, PC: MBS2601439, My BioSource). Immunoglobulin G, A, and M concentrations were determined using ELISA kits. Inflammatory cytokines, such as interferon-gamma (IFN-γ), tumor necrosis factor-α (TNF-α), and interleukin 4 (IL-4), were assessed in rabbit plasma using sandwich ELISA kits (IFN-γ: MBS2601171; TNF-α: MBS7612133; IL-4: MBS733925, My BioSource). Nitric oxide (NO) and Lysosome activity concentrations were determined following the methodologies outlined by [11,12] respectively. Amyloid A concentrations were evaluated using commercially available sandwich ELISA kits (Biosource). All laboratory analyses and biochemical evaluations were conducted in accordance with the protocols of ISO/IEC 17025, with the most recent version dating back to 2005.

### Histology

For histological examination, liver samples from both the control group and the EEONE treated groups were collected. The liver specimens were fixed in a 10% neutral formalin buffer and subjected to a dehydration process with increasing concentrations of ethyl alcohol, followed by clearing in xylol. Post-dehydration, the liver samples were embedded in paraffin blocks and sliced using a microtome into 4 μm sections (Leica RM 2155). These tissue sections were stained with eosin and hematoxylin for observation under a digital camera microscope (Leica DM 500, Leica EC3; Leica) to capture histological features. Histopathological features of hepatic steatosis were assessed using a semi-quantitative scoring system adapted from the recently established AASLD criteria for steatosis staging (Table 2).

### Transmission electron microscope

The liver samples were initially placed in cold 2.5% glutaraldehyde in 0.1 M phosphate buffer for 24 hours at 4°C, followed by washing and postfixation in 1% osmium tetroxide for 2 hours. After dehydration in a series of alcohol gradients (50%, 70%, 90%, 95%, and 100%), the samples were cleared with Acton and then embedded in epoxy resin. Subsequently, cut ultrathin sections (60–70 nm thick) were obtained using an ultramicrotome for further processing. Staining was conducted using lead citrate and uranyl acetate. The ultrathin liver tissue sections were examined under a TEM (TEM-JEOL 2100) operating at 160 kV.

### Statistical analysis

Data normality and homogeneity of error variances were evaluated using the Shapiro–Wilk test and Levene’s test, respectively. The MIXED procedure (PROC MIXED; SAS Institute) was utilized to evaluate various parameters, such as growth indices, feed utilization, carcass traits, and all blood hematological and biochemical parameters. In the statistical model, individual rabbits were considered as random factors, while EEONE concentrations were regarded as fixed factors. To determine significant differences between means, the Tukey test was employed in cases of significant effects of dietary treatment. A polynomial regression analysis was employed to examine the correlation between dietary levels of EEONE (0, 100, 200, and 400 mg/kg diet) and various parameters. Dose-response curves were generated using GraphPad Prism software ver. 9.0 (GraphPad).

## RESULTS

### Meteorological parameter

During the whole experimental period, the overall means of AT, RH, and temperature–humidity index were estimated to be 31.58±0.14°C, 70.01±0.59%, and 29.98±0.10 respectively. These calculated values indicate that the newly weaned rabbits were exposed to severe HS conditions.

### Characterization of eucalyptus essential oil nanoemulsion formation

**Figure 1:**
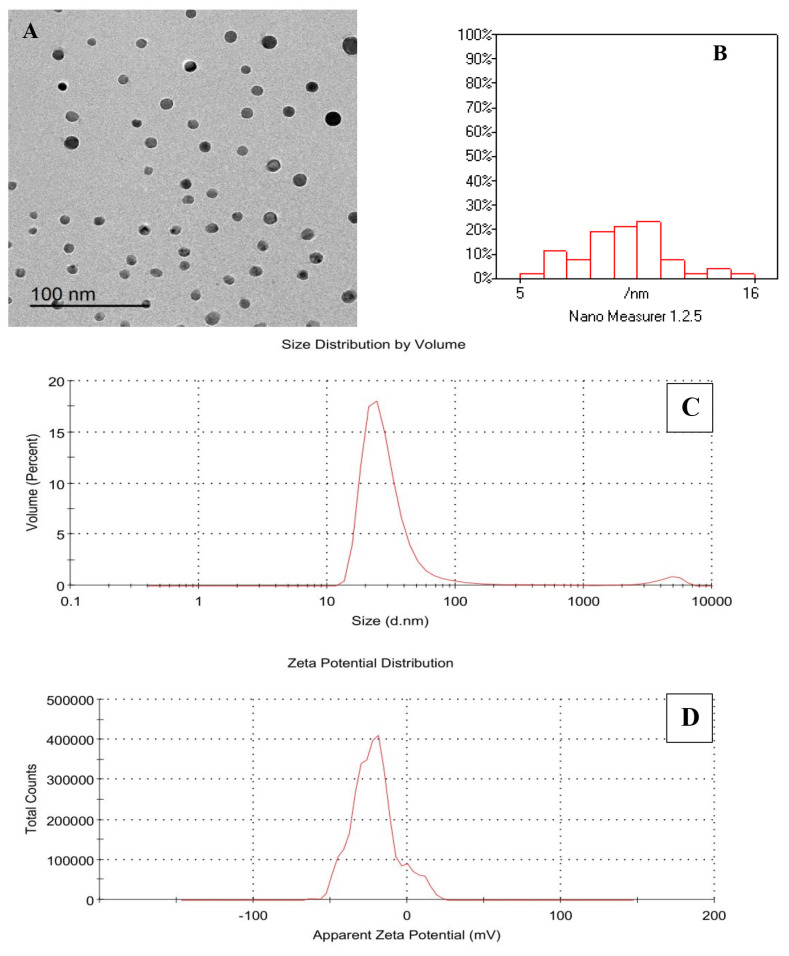
Morphological characterization of eucalyptus essential oil nanoemulsion (EEONE). (A) Transmission electron microscopy (TEM) image showing nearly spherical nanoparticles. (B) Particle size distribution histogram indicating that most particles range from 5 to 16 nm, demonstrating a uniform distribution. (C) Zetasizer analysis showing particle size distribution by volume. (D) Zeta potential distribution, indicating the stability of the nanoemulsion.

Figure 1A displays a transmission electron microscopy (TEM) image of EEONE, revealing revealed spherical and relatively uniform nanoparticle morphology. The average particle size ranged from 5 to 16 nm (Figure 1B). Dynamic light scattering analysis determined an average particle size of 97.35 nm with a PDI of 0.495 (Figure 1C). Zeta potential analysis indicated a negative surface charge of −21.7 mV (Figure 1D).

### Growth performance, feed utilization, and physiological responses

Table 3 illustrated the effect of dietary EEONE on growth performance and feed utilization. The average daily gain exhibited a significant linear increase with dietary treatment (p< 0.0001). Conversely, FCR decreased linearly in all treated groups compared to the control group (p = 0.0047). The performance index, representing the relationship between growth performance and feed utilization, linearly increased due to the dietary supplement (p = 0.0007). There were no significant differences in feed intake between the control group and all EEONE-treated groups (p = 0.3927). Regarding physiological responses, both rectal temperature and respiration rate decreased linearly in response to the dietary treatment, with no significant differences observed between the EEONE200 and EEONE400 treated groups (p>0.05; Table 3).

### Carcass traits

Results in Table 4 displays the carcass traits of heat-stressed rabbits received various levels of EEONE compared to control group. The liver’s relative weight was notably affected by the dietary treatment (p = 0.0417), showing a linear increase due to the dietary treatment (p = 0.0314). There were no significant differences between the control group and both the EEONE100 and EEOEN200 treated groups (p>0.05).

### Liver histological investigation

**Figure 2:**
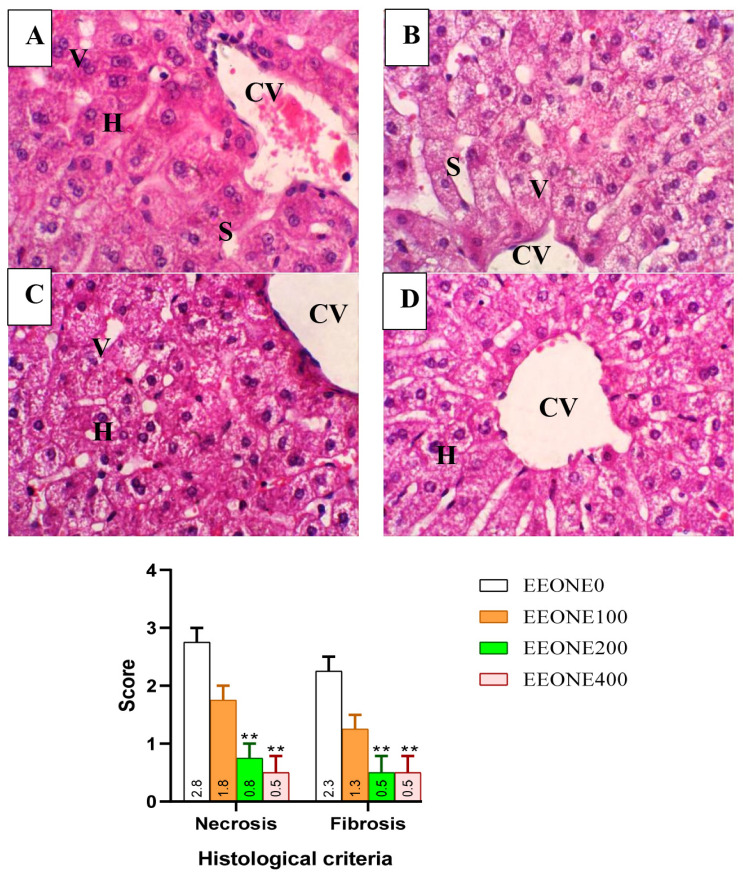
Photomicrograph of a paraffin section of the liver of the control under heat stress (A) and treated groups, including EEONE100 (B), EEONE200 (C), and EEONE400 groups (D). ** p<0.001 compared to the control group, as determined by t-test. V, vacuoles; CV, center vein; H, hepatocytes; S, sinusoid; EEONE, eucalyptus essential oil nanoemulsion (H&E, ×400).

In this study, the liver photomicrographs corroborated the findings from the blood profile and redox status analysis. The EEONE0 group exhibited minimal per portal vein, characterized by diffuse, apoptotic hepatocytes with intracellular vacuolation, necrotic nuclei, and focal per portal cellular infiltrates with small numbers of macrophages (Figure 2A). In contrast, the EEONE100 treated group displayed a nearly normal histological appearance of hepatic cells, with minimal micro vacuoles in hepatocytes (Figure 2B). However, the EEONE200 and EEONE400 treated groups showed normal hepato-portal structures with well-arranged hepatic parenchyma. The hepatic lobules were radially arranged around central veins, sinusoids were normal, and hepatocytes showed minimal cytoplasmic vacuolation (Figures 2C, 2D). Regarding the histopathological scoring of hepatic lesions, necrosis and fibrosis scores were significantly lower in both the EEONE200 and EEONE400 treated groups compared to the control group (p<0.001).

### Liver ultrastructure

**Figure 3:**
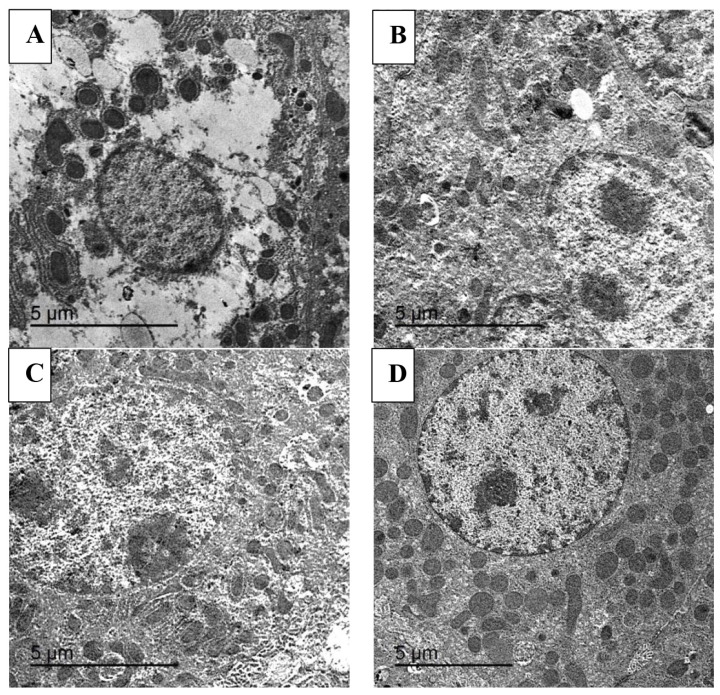
Representative photomicrograph of liver ultrastructure by TEM for the control under heat stress (A) and treated groups, including EEONE100 (B), EEONE200 (C), and EEONE400 groups (D). TEM, transmission electron microscope; EEONE, eucalyptus essential oil nanoemulsion.

Figure 3 illustrates the effect of dietary supplementation of EEONE on liver ultrastructure in rabbits exposed to serve HS. In the control group, liver cells exhibited abnormalities such as cytoplasmic vacuolation, irregular basement membrane, pyknosis nuclei, dilated rough endoplasmic reticulum, and some mitochondrial damage with irregular shapes and sizes (Figure 3A). In contrast, the EEONE100 group showed mostly normal cytoplasmic organelles with normal-looking nuclei and minimal vacuolation (Figure 3B). The EEONE200 group exhibited well-organized cytoplasmic organelles with some focal dilation in the rough endoplasmic reticulum and normal mitochondria and nuclei (Figure 3C). Regarding, the EEONE400 treated group, it showed hepatocytes with well-organized cytoplasmic organelles, dilated nuclei, and minimal cytoplasmic vacuolation (Figure 3D).

### Hematological attributes

**Figure 4:**
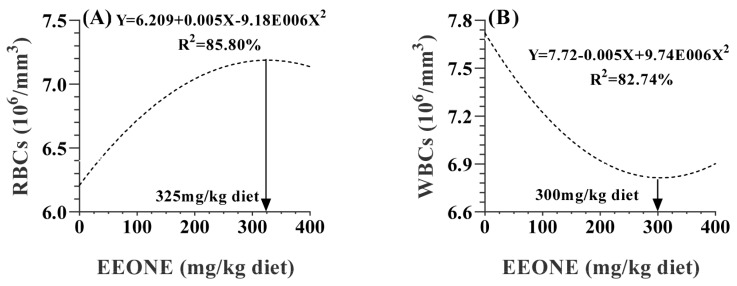
A polynomial regression analysis between dietary levels of EEONE and red blood corpuscles (RBCs; A), and white blood cell (WBCs; B). EEONE, eucalyptus essential oil nanoemulsion.

Table 5 presents the effect of EEONE dietary treatments on hematological parameters. Hemoglobin and platelet count values exhibited a significant linear increase due to the dietary treatment (p = 0.0291 and <0.0001, respectively). In terms of erythrocyte and leukocyte counts, red blood corpuscles (RBCs) displayed a quadratic increase (p = 0.0196), while white blood cell (WBCs) count showed a quadratic decrease (p = 0.0457) in response to EEONE treatments. The optimal doses were determined to be 325 mg EEONE/kg of diet for RBCs and 300 mg EEONE/kg of diet for WBCs (Figures 4A, 4B, respectively).

### Blood profile

**Figure 5:**
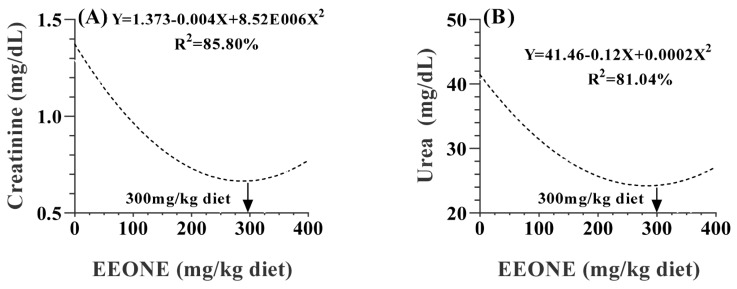
A polynomial regression analysis between dietary levels of EEONE and creatinine (A), and urea (B). EEONE, eucalyptus essential oil nanoemulsion.

In Table 5, most blood biochemical constituents were statistically affected by the dietary treatment (p<0.05 or <0.001). Serum levels of total protein and glucose decreased linearly in response to the EEONE treatment (p = 0.0150, 0.0233, respectively). Regarding the lipid profile, total cholesterol and triglycerides did not significantly affected by the dietary treatment (p = 0.9276 and 0.8974, respectively). In terms of kidney function, regression analysis revealed that serum creatinine and urea levels decreased quadratically due to EEONE treatment (p = 0.0025 and <0.0001, respectively), reaching a minimum level at the dose of 300 mg EEONE/kg of diet (Figures 5A, 5B).

### Redox status

**Figure 6:**
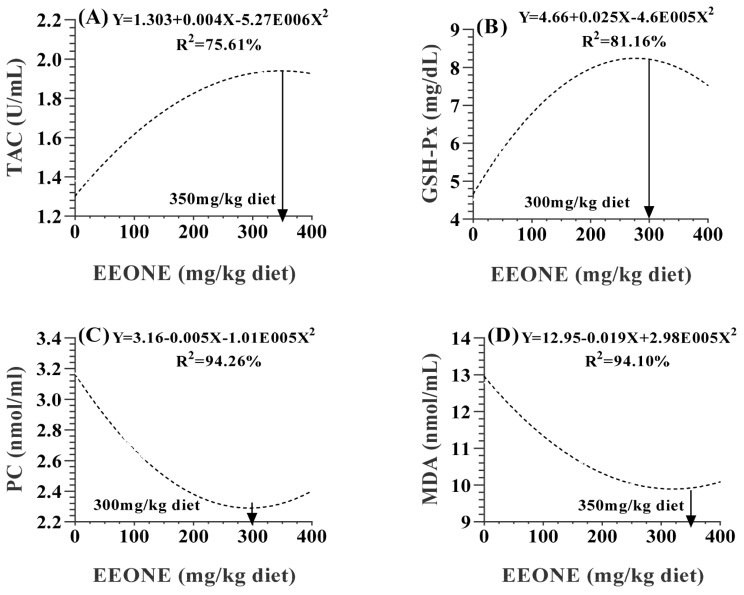
A polynomial regression analysis between dietary levels of EEONE and total antioxidant capacity (TAC; A), glutathione peroxidase (GSH-Px; B), protein carbonyl (PC; C) and malondialdehyde (MDA; D). EEONE, eucalyptus essential oil nanoemulsion.

In Table 6, the significant positive impact of dietary EEONE on antioxidant capacity are evident in various serum parameters. There were significant increases in serum TAC (p = 0.0053), SOD (p = 0.0001), GSH-Px (p = 0.0006). Meanwhile, there were significant decreases in the levels of PC (p = 0.0185), and MDA (p = 0.0418). In contrast, the dietary treatment had not significant effects on glutathione concentration (p = 0.6957). Polynomial regression analysis revealed quadratic relationships between all the mentioned antioxidant indices and the different levels of EEONE, except for a linear relationship detected with SOD (p = 0.0017). The optimal doses were observed to be 350 mg EEONE/kg of diet for TAC and MDA (Figures 6A, 6D) and 300 mg EEONE/kg of diet for GSH-Px and PC (Figures 6B, 6C).

### Immunity and inflammatory cytokines

**Figure 7:**
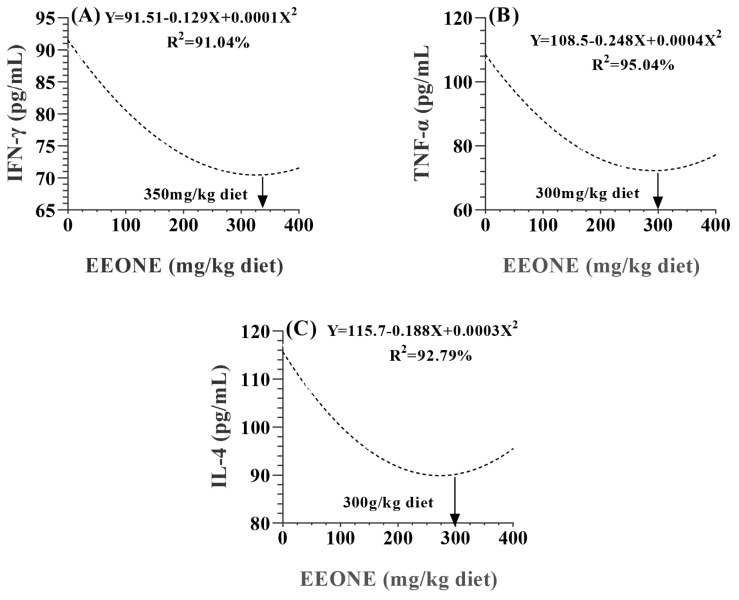
A polynomial regression analysis between dietary levels of EEONE and interferon-gamma (IFN-γ; A), tumor necrosis factors (TNF-α; B), and interleukin-4 (IL-4; C). EEONE, eucalyptus essential oil nanoemulsion.

With respected to the immunity status, results in Table 7 indicated that the dietary EEONE resulted in linear increase in levels of IgG, IgA, and IgM (p = 0.0002, 0.0105, and 0.0065, respectively). Also, NO concentration linearly increased by the dietary treatment (p = 0.0123). Regarding changes in inflammatory cytokines as a result of the EEONE treatment, interferon Y (IFN Y), TNF-α, and IL-4 exhibited quadratic decreases due to EEONE treatment, minimizing at doses of 350 mg EEONE/kg of diet for IFN-Y (Figure 7A) and 300 mg EEONE/kg of diet for both TNF-α and IL-4 (Figures 7B, 7C). Amyloid A and lysozyme activity showed linear improvements with the dietary treatment (p<0.0001), with no significant differences between the EEONE200 and EEONE400 treated groups (p>0.05).

## DISCUSSION

HS poses a severe challenge to the contemporary rabbit farming sector, particularly in tropical and subtropical areas. This stress stems from elevated ATs and humidity. These conditions trigger a cascade of unfavorable effects on blood chemistry, hormonal balance, immune responses, and antioxidant capacity, ultimately impacting production metrics like growth performance, carcass quality, and reproductive outcomes [2]. Implementing feeding strategies and nutritional adjustments shows promise in alleviating the detrimental effects of HS. Former studies suggests that enriching the diets of heat stressed rabbits with phytogenic additives, such as essential oils, could be a beneficial approach to mitigate the influence of elevated environmental temperatures. EEO offers a range of therapeutic benefits, encompassing antioxidant, antimicrobial anti-inflammatory, antiviral, and hepatoprotective properties [13]. These qualities make it a fitting dietary addition for newly weaned rabbits. However, despite the advantageous biological impacts of essential oils on animal well-being, their efficacy may be hindered by some challenges such as limited bioavailability and industrial constraints [14]. Since most essential oils are lipophilic molecules, their absorption in the gastrointestinal tract can be induced by presenting them in emulsified form. The slight negative charge, small size, and moderate hydrophilicity of nanoparticles facilitate their passage through the thin mucus layer of the small intestine [15]. Research reported that anionic nanoparticles can enhance the relaxation of tight junctions, thereby increasing intestinal permeability. This improvement in permeability is affected by the charge and size of the nanoparticles, with smaller particles (below 200 nm) and those with more negative charges exhibiting superior permeation capabilities [3]. In the current study, we employed a single-layer nanoemulsion method to create EEONE in nano-sized particles. The findings regarding the physicochemical characteristics such as a size of 5 to 16 nm validate the suitability of this nanoemulsion for improved cellular uptake and intestinal absorption. This approach promotes the bioavailability of the key constituents of EEONE, facilitating better utilization of its bioactive components.

The THI serves as a valuable tool for assessing the risk of HS faced by rabbits in tropical and subtropical regions [16]. Despite the THI values in this investigation signaling severe HS for the newly weaned rabbits [10], those supplemented with EEONE demonstrated enhanced heat tolerance, improved growth indices, redox balance, and overall health status. The incorporation of EEONE into the rabbit diets led to a notable reduction in body temperature. This suggests that the bioactive constituents found in EEONE, such as flavones and flavonoids, exhibit thermoregulatory effects [17]. Achieving thermoregulation necessitates a balance between heat dissipation and retention within the animal’s body. Consequently, under severe HS conditions, rabbits elevate their respiratory rate to release heat through evaporation via the respiratory system. This phenomenon elucidates the observed rise in breathing rate in the control group compared to the other EEONE-treated groups. [3] have substantiated the thermoregulatory impacts of certain essential oils in rabbits, attributed to their potent antioxidant activities.

The enhanced growth parameters seen in fattened rabbits receiving EEONE in their diets, especially at the levels of 200 or 400 mg/kg of diet, clearly indicate that EEONE may markedly enhance metabolic efficiency and nutrient absorption. These two treated groups showed average daily gains 97 g and 109.5 g higher than the control group, respectively, helping the rabbits better withstand the adverse effects of severe HS. With reduced FCRs of 0.28 and 0.53, respectively, these two treated groups suggested more efficient energy utilization, a crucial benefit under environmental stressors that can compromise feed intake and digestion [3]. These findings imply that dietary approaches enhancing energy provision and nutrient digestibility offer a practical means to effectively support growth and productivity in heat-stressed growing rabbits. Moreover, our results bolster growing evidence that nanoemulsified phytogenic additives not only enhance feed efficiency but also support thermoregulation, ultimately contributing to increased farm profitability during summer season.

Hematological parameters serve as valuable indicators of rabbits’ health status, immune function, susceptibility to infections, and environmental stressors. HS can disrupt these blood values, especially the levels of erythrocyte and leucocyte counts, rendering rabbits more vulnerable to illnesses. [17] noted that HS can reduce leucocyte levels, compromising immune function by affecting WBCs counts in fattened rabbits. The dietary addition of EEONE resulted in a notable increase in platelet counts, and RBCs as well as the concentration of hemoglobin. Additionally, dietary supplementation with EEONE quadratically reduced WBCs counts, maintaining them within the normal range, indicating an improved health status of growing rabbits exposed to elevated ATs.

Blood biochemical parameters serve as indicators of rabbits’ physiological responses to both internal and external conditions. While blood protein levels typically decrease during HS conditions [18], this study observed a significant increase in the levels of blood protein in rabbits supplemented with EEONE. This suggests an improved nutritional status in the EEONE-treated groups compared to the control group. With regard to blood glucose concentration, a notable rise is often seen in high-temperature conditions [18], likely attributed to reduced feed intake and subsequent metabolic rate decline. Furthermore, [19] demonstrated a direct link between elevated blood glucose levels and HS, primarily due to the release of glucocorticoids into the bloodstream. In this study, rabbits treated with EEONE200 and EEONE400 showed reductions in blood glucose concentrations by 14.9 and 16.1 mg/dL, respectively, compared to the control group. These findings align with [20], who noted that the inclusion of natural phytochemicals in the diets of heat-stressed growing rabbits led to a significant decrease in blood serum glucose levels.

Regarding carcass traits, the liver’s relative weight was notably higher in the EEOE400 treated group compared to the control group, confirming an enhancement in the health status of the fattened rabbits, given the liver’s pivotal role in synthesizing enzymes related to blood protein synthesis and heat tolerance [21]. Despite essential oils being rapidly metabolized, there is a potential risk of liver damage leading to increased enzyme secretion in the blood [22]. However, our study revealed lower serum levels of liver enzymes activities (AST and ALT) and reduced concentrations of sera urea and creatinine, suggesting that the administration of EEONE as a feed supplement was beneficial and safe in supporting liver and renal functions. In line with this investigation [23], reported that prolonged exposure to high concentrations of essential oils did not induce renal and/or nephritis failure. The current results are further supported by histopathological observations, which indicated a reduction in severe degenerative and necrotic changes in the livers tissues of rabbits exposed to HS following the dietary addition of EEONE. Additionally, the ultrastructure examination of liver sections revealed that the dietary supplement of 200 or 400 mg EEONE/kg diet led to healthier hepatocytes and nearly normal liver architecture compared to the control group. These findings align with the findings of [17] who demonstrated a significant restoration of hepatocyte ultrastructure in rabbits receiving medical herds for 14 weeks.

Under HS conditions, there is a notable generation of reactive oxygen species (ROS), leading to oxidative stress and impairing vital molecules like nucleic acids and proteins [24]. The antioxidant defense system, including enzymes like SOD and GSH-px, aims to balance ROS production. Former studies has shown that elevated ATs typically increase oxidative stress by promoting lipid and protein oxidation while disrupting the production of antioxidant enzymes [3,6,17]. In this study, the dietary incorporation of EEONE demonstrated improvements in anti-oxidative stress markers. The EEONE200 or EEONE400 treated groups exhibited higher levels of SOD, and GSH-px and lower levels of MDA and PC compared to the control group. Additionally, TAC increased by 52.30% and 42.30% in the EEONE200 and EEONE400 groups relative to the control group. These findings are in line with former studies suggesting that essential oils can mitigate the detrimental impacts of HS, improving oxidative stability by delaying the oxidation of proteins, lipids, and other nutrients and inhibiting oxidation reactions [6]. [5] indicated that TAC significantly induced with increasing concentrations of eucalyptus oil in the diets of Jabali and V-line rabbits. This improvement could be attributed to the presence of 1,8-cineole, polyphenols, and tannins in eucalyptus oil, which are known to boost antioxidant activity [25,26].

HS is known to generate free radicals, hydrogen peroxide, and pro-inflammatory cytokines, resulting in a notable impairment in immune function. This process is primarily mediated by the hypothalamic-pituitary-adrenal axis, as indicated by [2], which triggers glucocorticoid actions as part of an anti-immune response mechanism [27]. Elevated glucocorticoid levels can compromise both humoral and cellular immune responses. Consequently, HS involves in substantial losses in rabbit industry by impairing immune function, making rabbits more vulnerable to pathogens, as highlighted by [10]. In this study, significant linear increases (p<0.05) were observed in the levels of Immunoglobulin A, Immunoglobulin G, and Immunoglobulin M upon supplementing EEONE in rabbit diets, suggesting that EEONE contains bioactive compounds, particularly α-pinene and eucalyptol, known for their immune-stimulatory properties. Further, [28] elucidated that bioactive compounds or phytochemicals possess diverse pharmacological activities such as antimicrobial hepatoprotective, antioxidant, anti-helminthic, antiviral, and antifungal properties. Phenols, acting as immune boosters or antioxidants, play a crucial role in defending the body against pathogenic invasions [29]. The present outcomes are consistent with the results of [30], who noted a significant enhancement in cellular immunity in growing rabbits received diets containing varying levels of EEO. Additionally, supplementation of rabbit diets with EEONE led to notable improvements in humoral immunity, evident through increased amyloid A concentration and lysosomal activity. [31] noted that the heightened lysozyme activity may aid in pathogen elimination due to its enzymatic degradative potential, while amyloid A plays a crucial immunological role by stimulating cytokine synthesis or attracting neutrophils and mast cells [32]. These beneficial effects of essential oils on the immune system can be attributed to its broad biological activities, including anti-allergic, antiviral, antimicrobial, and anti-inflammatory properties, as highlighted by [33].

Elevated levels of pro-inflammatory cytokines like IFN-γ, TNF-α, and IL-4 are typically induced in the bloodstream under HS condition, leading to increased intestinal permeability to pathogens [34]. EEOs contain therapeutic compounds such as α-phellandrene, α-pinene, limonene, cineole, and terpineol, [35]. Limonene exhibits antinociceptive and antidepressant impacts, along with anti-inflammatory properties [36]. Eucalyptol (cineole) has been observed to inhibit pro-inflammatory molecules like TNF-γ and interleukin-1, as well as thromboxane and leukotrienes, crucial in the inflammation process [35]. The present study revealed quadratic significant reductions in the concentrations of pro-inflammatory cytokines (TNF-γ, IFN-α, and IL-4) in rabbits supplemented with EEONE compared to the control group, indicating the strong anti-inflammatory properties of EEONE. Furthermore, the dietary intervention resulted in a noteworthy increase in the levels of NO, which plays a vital role in defense against pathogens and thermo tolerance during HS conditions by inducing vasodilation of skin blood vessels [37]. Additionally, NO is implicated in neurotransmission and immune regulation [38]. The observed elevation in NO concentration in the blood serum of EEONE-supplemented rabbits, alongside increased antioxidant indices (GSH, CAT, SOD, and TAC), suggests that the NO levels were within a normal physiological range, given its classification as a free radical.

The present study highlights the positive impact of EEONE in mitigating HS in growing rabbits; however, it is limited by the absence of in-depth molecular understanding of its of its antioxidant and immunomodulatory mechanisms. Although nanoemulsification enhanced the bioavailability of EEO, additional pharmacokinetic investigations are necessary to fully elucidate its metabolism, absorption, and excretion. Moreover, because this study was limited to a single rabbit breed in specific environmental conditions, more studies involving diverse breeds and settings are needed to confirm and generalize these results. Future studies should aim to investigate the molecular mechanisms involved, carry out long-term evaluations of health and reproductive outcomes, and exploring possible synergistic interactions with other medical herbs to enhance resilience to HS.

## CONCLUSION

Supplementing the diets of heat-stressed growing rabbits with phytogenic antioxidants sourced from EEONE can alleviate the negative effect of HS on feed efficiency, immune response, antioxidant levels, and pro-inflammatory cytokines, ultimately enhancing their growth performance and overall health. Through dose-response analysis, it was obtained that a dosage range of 300–400 mg EEONE/kg diet is optimal for enhancing the growth indices and well-being of growing rabbits facing serve HS conditions. Additional mechanistic studies are needed to elucidate the specific pathways through which EEONE exerts its effects in rabbits.

## Figures and Tables

**Table 1:** Ingredients and chemical composition of the experimental basal diet

Items	%
Ingredients (%)	
Alfalfa hay	31.00
Barley grain	24.60
Wheat bran	28.00
Soybean meal	13.25
Di-calcium phosphate	1.60
Mineral-vitamin premix^1)^	0.30
Sodium chloride	0.30
Limestone	0.95
Total	100
Chemical analysis (% on dry matter basis)	
Crude protein (CP, %)	17.08
Ether extract (EE, %)	2.20
Crude fiber (CF, %)	12.55
Methionine (%)	0.23
Calcium (%)	1.20
Lysine (%)	0.84
Total phosphorus (%)	0.761
Digestible energy (DE, kcal/kg)	2,416
Metabolizable energy (ME, kcal/kg)	2,219

1 kilogram of minerals–vitamins premix provided as: Niacin, 200 mg; Folic acid, 10 mg; Pantothenic acid, 100 mg; Vitamin E, A, and K3 (100 mg, 150,000 IU, and 21 mg); Vitamin B1, B6, and B12 (10, 15, and 0.1 mg); Choline chloride, 5,000 mg; Biotin, 0.5 mg; Cu, 50 mg; Mn, 600 mg; Fe, 0.3 mg; Se, 1 mg; Zn, 450 mg; and Co, 2 mg.

**Table 2:** Histopathological scoring of hepatic lesions

Histological criteria	Severity	Score
Necrosis	Absent	0
	Mild	1
	Marked	2
	Severe	3
Fibrosis	Absent	0
	Mild	1
	Marked	2
	Severe	3

**Table 3:** Growth performance, feed utilization, and physiological response of heat stressed rabbits fed diet enriched with 0 (EEONE0), 100 (EEONE100), 200 (EEONE200), and 400 (EEONE400) mg EEONE/kg diet

Items	Treatments (TRTs)				SEM	p-values			
	
	EEONE0	EEONE100	EEONE200	EEONE400		TRTs	Lin.	Quad.	Cub.
IBW (g)	698.30	690.00	690.10	701.00	6.000	0.4571	0.7617	0.1184	0.9292
FBW (g)	2,003.60^b^	2,087.30^a^	2,100.60^a^	2,113.10^a^	16.719	<0.0001	<0.0001	0.0708	0.2974
ADG (g)	23.31^b^	24.95^a^	25.19^a^	25.22^a^	0.250	<0.0001	<0.0001	0.0827	0.2903
FI (g)	111.60	113.50	113.40	107.40	2.817	0.3927	0.3202	0.1695	0.7587
FCR	4.79^a^	4.55^ab^	4.51^ab^	4.26^b^	0.121	0.0344	0.0047	0.9702	0.4584
PI (%)	42.00^b^	46.00^ab^	47.56^a^	49.70^a^	1.486	0.0066	0.0007	0.5367	0.6524
Physiological response									
RR	144.26^a^	137.26^b^	133.26^bc^	131.34^c^	0.927	0.0039	0.0137	0.0964	0.4138
RT	39.03^a^	38.44^b^	38.36^b^	38.29^b^	0.011	0.0001	0.0024	0.3261	0.2284

a–c Means in the same row with different superscript letter following them are significantly different (p<0.05).

EEONE, eucalyptus essential oil nanoemulsion; Lin, linear; Quad, quadratic; Cub, cubic; IBW, initial body weight; FBW, final body weight; ADG, average daily gain; FI, feed intake; FCR, feed conversion ratio; PI, performance index; RR, respiration rate; RT, rectal temperature.

**Table 4:** Carcass traits of heat stressed rabbits fed diet enriched with 0 (EEONE0), 100 (EEONE100), 200 (EEONE200), and 400 (EEONE400) mg EEONE/kg diet

Items	Treatments (TRTs)				SEM	p-values			
	
	EEONE0	EEONE100	EEONE200	EEONE400		TRTs	Lin.	Quad.	Cub.
LBW	2,088.20	2,074.33	2,076.20	2,072.11	31.896	0.9841	0.7536	0.8833	0.8866
Carcass traits (as % of live weight)									
Dressing	53.42	55.54	56.31	56.05	1.056	0.2759	0.1041	0.2916	0.9434
Head	5.31	5.37	5.15	5.42	0.396	0.9646	0.9566	0.7925	0.6766
Liver	3.07^b^	3.11^ab^	3.17^ab^	3.26^a^	0.009	0.0417	0.0314	0.9304	0.7982
Heart	0.37	0.30	0.29	0.28	0.039	0.4754	0.1824	0.5148	0.7064
Lung	0.70	0.65	0.74	0.71	0.097	0.9299	0.7878	0.9658	0.5671
Kidney	0.65	0.61	0.67	0.64	0.066	0.9522	0.9207	0.9205	0.5946
Spleen	0.10	0.10	0.11	0.12	0.020	0.9076	0.5231	0.8863	0.7983

a,b Means in the same row with different superscript letter following them are significantly different (p<0.05).

EEONE, eucalyptus essential oil nanoemulsion; Lin, linear; Quad, quadratic; Cub, cubic; LBW, live body weight.

**Table 5:** Blood hematology and biochemical constituents of heat stressed rabbits fed diet enriched with 0 (EEONE0), 100 (EEONE100), 200 (EEONE200), and 400 (EEONE400) mg EEONE/kg diet

Items	Treatments (TRTs)				SEM	p-values			
	
	EEON0	EEONE100	EEONE200	EEONE400		TRTs	Lin.	Quad.	Cub.
Blood hemtology									
Hb (g/dL)	11.43^b^	13.90^ab^	13.91^ab^	14.19^a^	0.476	0.0461	0.0291	0.2327	0.4358
RBCs (10^6^/mm^3^)	6.21^b^	6.43^b^	7.18^a^	7.06^a^	0.137	0.0094	0.0820	0.0196	0.2528
WBCs (10^3^/mm^3^)	7.72^a^	7.40^ab^	6.80^ab^	6.72^b^	0.260	0.0314	0.1664	0.0457	0.2382
PLT (10^3^/mm^3^)	274.16^c^	277.78^c^	299.59^b^	318.80^a^	4.468	<0.0001	<0.0001	0.1063	0.3186
HCT (%)	31.48	31.25	30.18	31.30	2.251	0.9755	0.8752	0.7686	0.7694
Blood biochemical									
TP (g/dL)	6.38^b^	7.06^a^	7.10^a^	7.18^a^	0.103	0.0065	0.0150	0.4329	0.6703
Glob (g/dL)	2.71	2.94	3.03	3.28	0.163	0.5160	0.1519	0.9629	0.8030
Alb (g/dL)	3.67	4.12	4.07	3.90	0.128	0.4589	0.9321	0.1281	0.7659
Glu (mg/dL)	109.26^a^	101.25^ab^	94.36^ab^	93.16^b^	1.751	0.0152	0.0233	0.4870	0.8340
TC (mg/dL)	175.16	179.00	171.51	172.07	8.880	0.9276	0.6807	0.8568	0.6340
TG (mg/dL)	74.28	71.32	71.95	75.44	4.384	0.8974	0.8369	0.4762	0.9701
Creat (mg/dL)	1.37^a^	1.11^a^	0.63^b^	0.78^b^	0.085	0.0181	0.0025	0.0408	0.8868
Urea (mg/dL)	41.46^a^	30.22^b^	27.65^b^	29.24^b^	1.204	<0.0001	<0.0001	0.0331	0.7509
AST (IU/L)	36.81^a^	31.30^ab^	30.00^b^	28.04^b^	2.103	0.0422	0.0125	0.4144	0.6143
ALT (IU/L)	60.75^a^	56.83^ab^	55.13^ab^	51.33^b^	2.613	0.0366	0.0249	0.9813	0.7187

a–c Means in the same row with different superscript letter following them are significantly different (p<0.05).

EEONE, eucalyptus essential oil nanoemulsion; Lin, linear; Quad, quadratic; Cub, cubic; Hb, hemoglobin; RBCs, red blood corpuscles; WBCs, white blood cell; PLT, platelet count; HCT, hematocrit; TP, Total protein; Glob, globulin; Alb, albumin; Glu, glucose; TC, total cholesterol; TG, triglyceride; Creat, creatinine; AST, aspartate aminotransferase; ALT, alanine aminotransferase.

**Table 6:** Redox status of heat stressed rabbits fed diet enriched with 0 (EEONE0), 100 (EEONE100), 200 (EEONE200), and 400 (EEONE400) mg EEONE/kg diet

Items	Treatments (TRTs)				SEM	p-values			
	
	EEONE0	EEONE100	EEONE200	EEONE400		TRTs	Lin.	Quad.	Cub.
TAC (U/mL)	1.30^b^	1.41^b^	1.98^a^	1.75^a^	0.113	0.0053	0.0064	0.0130	0.3093
SOD (ng/mL)	1.30^b^	1.54^b^	1.58^b^	2.32^a^	0.135	0.0001	0.0017	0.4481	0.4792
GSH (mg/dL)	16.11	16.51	16.27	17.27	0.729	0.6957	0.3425	0.6931	0.5762
GSH-Px (mg/dL)	4.66^b^	7.22^a^	7.64^a^	7.18^a^	0.332	0.0006	0.0080	0.0430	0.0491
PC (nmol/mL)	3.16^a^	2.66^b^	2.39^b^	2.40^b^	0.126	0.0185	0.0026	0.0435	0.4494
MDA (nmol/mL)	12.95^a^	11.77^ab^	10.00^b^	10.04^b^	0.638	0.0418	0.0148	0.0305	0.4375

a,b Means in the same row with different superscript letter following them are significantly different (p<0.05).

EEONE, eucalyptus essential oil nanoemulsion; Lin, linear; Quad, quadratic; Cub, cubic; TAC, total antioxidant capacity; SOD, superoxide dismutase; GSH, glutathione; GSH-Px, glutathione peroxidase; PC, protein carbonyl; MDA, malondialdehyde.

**Table 7:** Immunity and inflammatory cytokines of heat stressed rabbits fed diet enriched with 0 (EEONE0), 100 (EEON100), 200 (EEONE200), and 400 (EEONE400) mg EEONE/kg diet

Items	Treatments (TRTs)				SEM	p-values			
	
	EEOE0	EEOE100	EEOE200	EEOE400		TRTs	Lin.	Quad.	Cub.
IgA (ng/mL)	21.25^b^	25.33^ab^	26.83^ab^	27.02^a^	0.704	0.0059	0.0105	0.4131	0.9713
IgG (ng/mL)	53.21^b^	55.10^b^	62.80^a^	65.46^a^	1.890	0.0015	0.0002	0.8437	0.2235
IgM (mg/dL)	69.28^b^	83.30^a^	84.06^a^	86.79^a^	3.622	0.0214	0.0065	0.1452	0.3656
NO (Umol/L)	46.27^b^	47.98^ab^	50.53^b^	57.70^a^	2.412	0.0270	0.0123	0.2796	0.1024
IFN-γ (pg/mL)	91.51^a^	84.31^a^	70.80^b^	72.00^b^	2.124	0.0002	0.0005	0.0103	0.0629
TNF-α (pg/mL	108.53^a^	92.54^b^	72.48^c^	77.73^c^	4.465	0.0014	0.0022	0.0062	0.0621
IL-4 (pg/mL)	115.73^a^	96.38^b^	94.65^b^	95.00^b^	3.132	0.0010	0.0004	0.0183	0.2502
Amyloid A (ng/mL)	3.63^a^	2.69^b^	2.50^bc^	2.17^c^	0.143	<0.0001	<0.0001	0.0728	0.5048
LZM (ng/mL)	2.69^c^	2.79^b^	3.27^a^	3.51^a^	0.118	<0.0001	<0.0001	0.3723	0.6266

a–c Means in the same row with different superscript letter following them are significantly different (p<0.05).

EEONE, eucalyptus essential oil nanoemulsion; Lin, linear; Quad, quadratic; Cub, cubic; IgA, immunoglobulin A; IgG, immunoglobulin G; IgM, immunoglobulin M; NO, nitric oxide; IFN-γ, interferon-gamma; TNF-α, tumor necrosis factors; IL-4, interleukin-4; LZM, lysozyme.
